# Supplementary material for: Paradoxical SERCA2a Dysregulation Contributes to Atrial Fibrillation in a Model of Diet-Induced Obesity
Source: Int J Mol Sci. 2025 Jun 11;26(12):5603. doi: 10.3390/ijms26125603 (PMC12192961; doi:10.3390/ijms26125603)
Supplement: Supplementary file 1 [file ijms-26-05603-s001.zip › ijms-3618162-supplementary.pdf]

Supporting Information for

Paradoxical SERCA2a Dysregulation Contributes to Atrial Fibrillation in a Model of Diet-Induced Obesity

This PDF file includes:

Extended methods
Tables S1 to S4
Uncropped images of Western blots

Extended methods

Histology. Blood was removed by right ventricular puncture and the vasculature was perfused with ice-cold PBS. The heart was harvested, and isolated RA and LA were fixed in 4% paraformaldehyde, embedded in paraffin, and cut into 5 mm sections. Masson trichrome staining was used to evaluate interstitial fibrosis. Hematoxylin and eosin staining were used to evaluate fat accumulation. Paraffin-embedded sections of the fat pads were deparaffinized and rehydrated. After blocking, sections (6μm each) were incubated at room temperature for 2 hours with Mac2 (sc-81728; Santa Cruz Biotechnology Inc.). Mac2 slides were counterstained with hematoxylin and cover-slipped. Images were captured with an Olympus LC30 camera mounted on an Olympus CX41 microscope. For the $Mac2^+$ area, all images were obtained with the same light source at the same time. The $Mac2^+$ area was determined using the threshold function in ImageJ (NIH) and normalized to the total region of interest area. Results are reported as the percentage of adipose area. Sectioning and staining were performed by the In Vivo Animal Core Laboratory technicians at the Unit for Laboratory Animal Medicine, University of Michigan. Technicians in this laboratory were blinded to experimental identity.

Isolation of cardiac myocytes. Mice cardiac myocytes were isolated using a modified Langendorff technique. Briefly, mice were intraperitoneal injected with 0.1 mL heparin (1000 IU/mL) 20 min before heart excision. Animals were anesthetized with 2% isoflurane and the heart was removed quickly from the chest and retrogradely perfused through the aorta at a constant flow (4 mL/min) at 37 ^{0}C for 4 min with a Ca^{2+}-free buffer containing 113 mM NaCl, 4.7 mM KCl, 1.2 mM $MgSO_4$, 0.6 mM Na_2HPO_4, 0.6 mM KH_2PO4, 10 mM $KHCO_3$, 12 mM $NaHCO_3$, 10 mM HEPES, 10 mM 2,3-butanedione monoxime (BDM, sigma) 30 mM taurine, and 5.5 mM glucose. All solutions were filtered (0.2 mm filter) and equilibrated with O_2 (100%) for at least 20 min before use. Enzymatic digestion was initiated by adding collagenase type II (773.4 U/mL; Worthington), trypsin (0.14 mg/mL), and $CaCl_2$ (12.5 mM) to the perfusion solution. After 5-6 min of digestion, the atrial tissue was removed and placed in a well of a 24-well plate with enzymatic solution, stirred gently with a magnetic bar for 5 minutes. After this time the isolated atrial myocytes were collected, and the remaining tissue was transferred to another well containing enzymatic solution. This was repeated until the tissue was completely dissociated.

Measurement of I_{Ca} and I_K currents. For I_{Ca} measurements, we used the following pipette filing solution: 120 mM CsCl, 20 mM TEA-Cl, 1 mM $MgCl_2$, 5 mM MgATP, 0.2 mM Na_2GTP, 10 mM HEPES, and 10 mM EGTA, pH adjusted to 7.2 with CsOH. The external solution contained 137 mM NaCl, 5.4 mM CsCl, 1 mM $MgCl_2$, 1 mM $CaCl_2$, 10 mM HEPES, 10 mM glucose, 2 mM 4-aminopyridine (4-AP), and 10 mM tetrodotoxin (TTX); pH was adjusted to 7.4 with NaOH. Voltage dependence of peak I_{CaL} was measured by holding at -50 mV; 300 ms voltage steps were applied from -40 to +60 mV in 5 mV increments. The interval between voltage steps was 350 ms. For I_K measurements, we used the following pipette filling solution: 150 mM KCl, 1 $MgCl_2$, mM 5 EGTA, 5 mM HEPES, 5 mM phosphocreatine, 4.4 mM K_2ATP, 2 mM β-hydroxybutyric acid, pH adjusted to 7.2 with KOH. The external solution contained 148 mM NaCl, 0.4 mM NaH_2PO_4, 1 mM $MgCl_2$, 5.5 mM glucose, 5.4 mM KCl, 1 mM $CaCl_2$, and 15 mM HEPES; the pH was adjusted to 7.4 with NaOH. To measure I_K currents, we used 10 mM TTX and 5 mM nifedipine to block the fast voltage-gated Na^+ channels and the L-type Ca^{2+} currents, respectively. K^+ currents were recorded using 5-second depolarizing pulses to potentials between -120 mV to +50 mV from a holding of -70 mV. Voltage steps were in steps of 10 mV at 10-second intervals. Cells were constantly perfused with the appropriate external solution. All membrane currents were examined as current densities by normalizing to cell capacitance. For acute adrenergic stimulation, we used 10 μM isoproterenol.

Preparation of atrial myocytes for Ca^{2+} imaging studies. After myocyte dissociation, calcium reintroduction was gradually performed to a final concentration of 1 mM. The myocytes were allowed to equilibrate at room temperature for 10 min and transferred to a plating media solution with the following composition: culture media (see below) supplemented with 5% Fetal bovine serum (Sigma, F2442) and 25 μM S-(-) Blebbistatin (Sigma, B0560). Myocytes were counted using a hemocytometer, and approximately 20,000 cells were plated on previously 22x22 mm glass coverslips coated with laminin (40 μg/mL) using 6-well plates. The cells were allowed to attach for

40 min at room temperature. After cell attachment, the plating media was replaced with culture media with the following composition: 150 mL MEM +Hank's salts (Gibco, 500 mL 11575-032), 0.1% ITS liquid media supplement (100x, Sigma, I3146), 10 U/mL Penicillin/ 10 μg/mL Streptomycin (Invitrogen, 15070-063), 25 mM NaHCO3 (Sigma, S8875), 15 mM HEPES (Sigma H7006), 5 mM butanedione monoxime (BDM, sigma), and the cells were allowed to equilibrate for 20 min at room temperature.

Table S1. Results from the transcriptomics analysis show genes that are upregulated in the atria of mice fed a high-fat diet.

Gene ID	Description	log_2(FoldChange)	P_{adj}
Nnat	Neuronatin	3.9358749	3.75E-02
Sh3gl2	SH3-domain GRB2-like 2	1.6766716	3.67E-02
Acta2	Actin α-2	1.6285951	1.51E-03
Uchl1	Ubiquitin carboxy-terminal hydrolase L1	1.6152538	2.66E-02
Angptl4	Angiopoietin-like 4	1.5013384	3.55E-02
Slc25a34	Solute carrier family 25, member 34	1.3218376	1.17E-02
Cidea	Cell death-inducing DNA fragmentation factor	1.2472558	5.29E-05
Egln3	egl-9 family hypoxia-inducible factor 3	1.1866723	1.22E-04
Scd4	Stearoyl-coenzyme A desaturase 4	1.1364512	1.60E-03
Klhdc8a	Kelch domain-containing 8A	1.0969114	1.60E-03
Ech1	Enoyl coenzyme A hydratase 1	0.9524622	3.45E-14
Lipe	Lipase, hormone-sensitive	0.8801781	1.22E-02
Myl9	Myosin, light polypeptide 9	0.854425	7.00E-04
Notch3	Notch receptor 3	0.8333349	1.78E-02
Plin5	Perilipin 5	0.7815313	4.34E-02
Decr1	Mitochondrial 2,4-dienoyl CoA reductase 1	0.7790149	7.42E-09
Hspb6	Heat shock protein, α-crystallin-related, B6	0.7380947	7.27E-04
Mgll	Monoglyceride lipase	0.7357712	3.92E-03
Slc25a20	Solute carrier family member 20	0.6602545	4.78E-04
Ucp2	Uncoupling protein 2	0.6542212	9.06E-03
Vwa8	Von Willebrand Factor A Domain Containing 8	0.635321	5.29E-05
Hadha	α-subunit, mitochondrial trifunctional protein	0.6298784	5.29E-05
Cpt2	Carnitine palmitoyltransferase 2	0.6259808	9.94E-04
Acadvl	Acyl-Coenzyme A dehydrogenase, long chain	0.5910815	2.94E-04

Table S2. Results from the transcriptomics analysis show genes that are downregulated in the atria of mice fed a high-fat diet.

Gene ID	Description	log_2(FoldChange)	P_{adj}
Hao1	Hydroxyacid oxidase 1, liver	-4.56117892	2.59E-02
2810007J24RIK	Non-coding RNA lncLSTR	-3.32859513	2.77E-02
C8a	Complement component 8,α-polypeptide	-3.18667035	4.61E-02
Mup12	Major urinary protein 12	-3.08277672	3.00E-02
Ugt2b36	UDP glucuronosyltransferase 2, PP 36	-2.79395868	5.35E-04
Aldob	Aldolase B, fructose-bisphosphate	-2.74285967	3.29E-02
Cth	Cystathionase (cystathionine γ-lyase)	-2.64470838	4.61E-02
Fga	Fibrinogen α-chain	-2.60214385	6.01E-03
Cyp2c29	Cytochrome P450, family 2, PP29	-2.58927548	7.70E-04
Pigr	Polymeric immunoglobulin receptor	-2.53607478	1.03E-02
Serpina1a	Serine/Cysteine peptidase inhibitor 1A	-2.53323819	3.64E-03
Hc	Hemolytic complement	-2.53075027	1.42E-02
Rdh7	Retinol dehydrogenase 7	-2.49011343	4.61E-02
G6pc	Glucose-6-phosphatase	-2.48081814	7.64E-03
Kng1	Kininogen 1	-2.47785248	2.66E-02
Hrg	Histidine-rich glycoprotein	-2.44995622	1.97E-02
Ugt2b1	UDP glucuronosyltransferase 2, PP B1	-2.43224654	3.30E-02
Arg1	Arginase	-2.41928113	2.59E-02
Alb	Albumin	-2.39526486	4.21E-03
Cyp3a11	Cytochrome P450, family 3, PP11	-2.39243285	3.10E-02
Fabp1	Fatty acid binding protein 1	-2.35790185	1.21E-02
Tdo2	Tryptophan 2,3-dioxygenase	-2.35455506	2.78E-04
Mup3	Major urinary protein 3	-2.33196502	1.10E-02
Fbp1	Fructose bisphosphatase 1	-2.32152002	3.92E-03
Slc27a2	Solute carrier family 27 member 2	-2.31684202	2.89E-03
Cyp2c70	Cytochrome P450, family 2, PP70	-2.06324976	4.74E-02
Gc	GC Vitamin D Binding Protein	-2.06264149	5.41E-04
Serpina1d	Serine/Cysteine peptidase inhibitor 1D	-2.0567446	1.99E-02
Uox	Urate oxidase	-2.04854547	2.65E-02
Rgn	Regucalcin	-1.93700546	4.71E-02
Gnmt	Glycine N-methyltransferase	-1.87548375	8.05E-03
Asgr1	Asialoglycoprotein receptor 1	-1.85369659	4.71E-02
Onecut2	One-cut domain, family member 2	-1.7671305	2.66E-02
Hpx	Hemopexin	-1.76009049	2.51E-02
Fam107a	Family with seq. Similarity 107, member A	-1.71388062	7.51E-03
Cyp2f2	Cytochrome P450, family 2, subfamily F, PP2	-1.68980162	2.49E-02
Pzp	PZP, α-2-macroglobulin like	-1.46863366	4.52E-03
Igfbp3	Insulin-like growth factor binding protein 3	-1.301052	5.29E-05
Apoc1	Apolipoprotein C-I	-1.29237357	2.44E-02
Irx5	Iroquois homeobox 5	-0.76118912	4.44E-02
Ahsg	α-2-HS-glycoprotein	-0.74651702	7.00E-04
Klf15	Kruppel-like factor 15	-0.74499343	2.74E-03

Table S3. Summary of gene expression of calcium-handling, sodium-transporting, and SERCA2a-regulating proteins in atria of mice fed a high-fat diet.

**Gene ID**	**Description**	**log_2(FoldChange)**	**P_{adj}**
Atp2a2	Sarcoplasmic reticulum Ca^{2+}-ATPase isoform 2a (SERCA2a)	-0.09544436	0.011429456
Pln	Phospholamban	-0.46219364	1.786313278
Ryr2	Cardiac ryanodine receptor 2 (RyR2)	0.1153773	0.014622124
Cacna1c	$Ca_v1.2$ L-type voltage-gated calcium channel	-0.07287843	0.004578043
Slc8a1	Na^+/Ca^{2+} exchanger protein 1	0.2411005	0.147312776
Casq2	Calsequestrin 2	0.2015695	0.248789215
Sln	Sarcolipin	-0.24274848	0.398687932
Atp1a1	Na^+/K^+ ATPase transporting subunit alpha 1 (NKA-α1)	0.2054246	0.045051404
Atp1a2	Na^+/K^+ ATPase transporting subunit alpha 2 (NKA-α2)	0.01571423	0.000645076
Fxyd1	Phospholemman	0.1064471	0.009333479
Fxyd2	FXYD Domain-Containing Ion Transport Regulator 2	1.0412618	0.072278579
Fxyd5	Dysadherin	0.4837579	0.446589599
Fxyd6	FXYD Domain-Containing Ion Transport Regulator 6	0.4823985	0.860362129
Fxyd7	FXYD Domain-Containing Ion Transport Regulator 7	-0.13341468	0.000645076
Scn5a	Sodium Voltage-Gated Channel Alpha Subunit 5 ($Na_v1.5$)	0.2409932	0.117074975
Sirt1	Sirtuin 1	-0.224188348	0.854507362
Sumo1	Small Ubiquitin Like Modifier 1	0.078081544	0.989338099

Table S4. Antibodies used to detect and quantify the expression of proteins in atrial tissue

**Primary antibodies**	**Vendor/Supplier**	**Dilution**
GAPDH	Sigma	1:2000
Serca2a	ThermoFisher	1:500
α-actinin	Sigma	1:1000
Phospholamban (PLN)	Badrilla	1:5000
Phosphorylated PLN (Ser16)	Badrilla	1:1000
Ryanodine Receptor (all RYR)	ThermoFisher	1:500
$Ca_v1.2$	Alomone	1:250
NCX1	Swant	1:500
**Secondary antibodies**		
Goat anti-mouse-HRP	Jackson Immunoresearch	1:1000<br>1:5000 for GAPDH
Goat anti-rabbit-HRP	Jackson Immunoresearch	1:1000

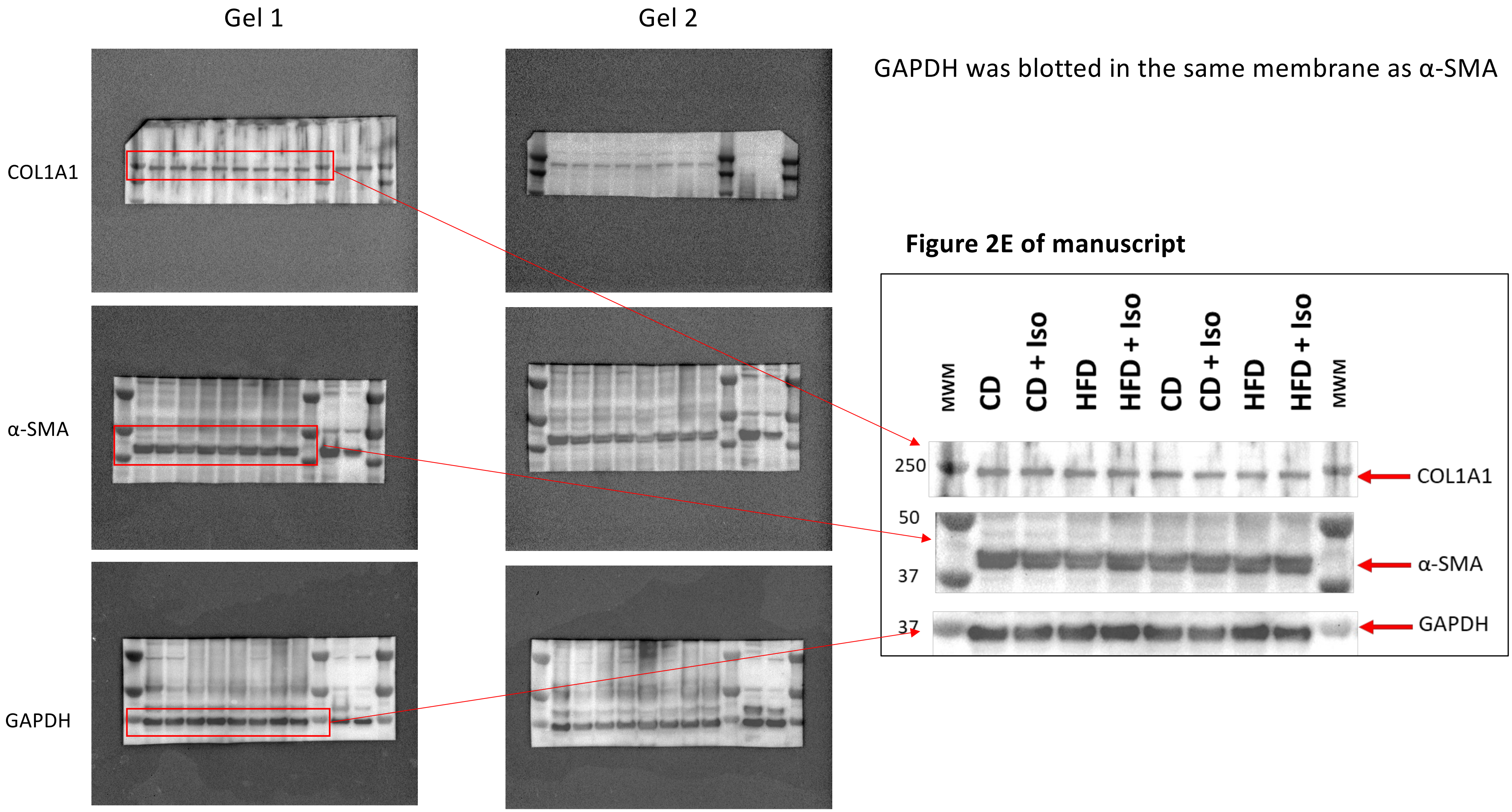
Gel 1
Gel 2
COL1A1
α-SMA
GAPDH
GAPDH was blotted in the same membrane as α-SMA
Figure 2E of manuscript
MWM
CD
CD + Iso
HFD
HFD + Iso
CD
CD + Iso
HFD
HFD + Iso
MWM
250
50
37
37
COL1A1
α-SMA
GAPDH

Figure 8A of manuscript

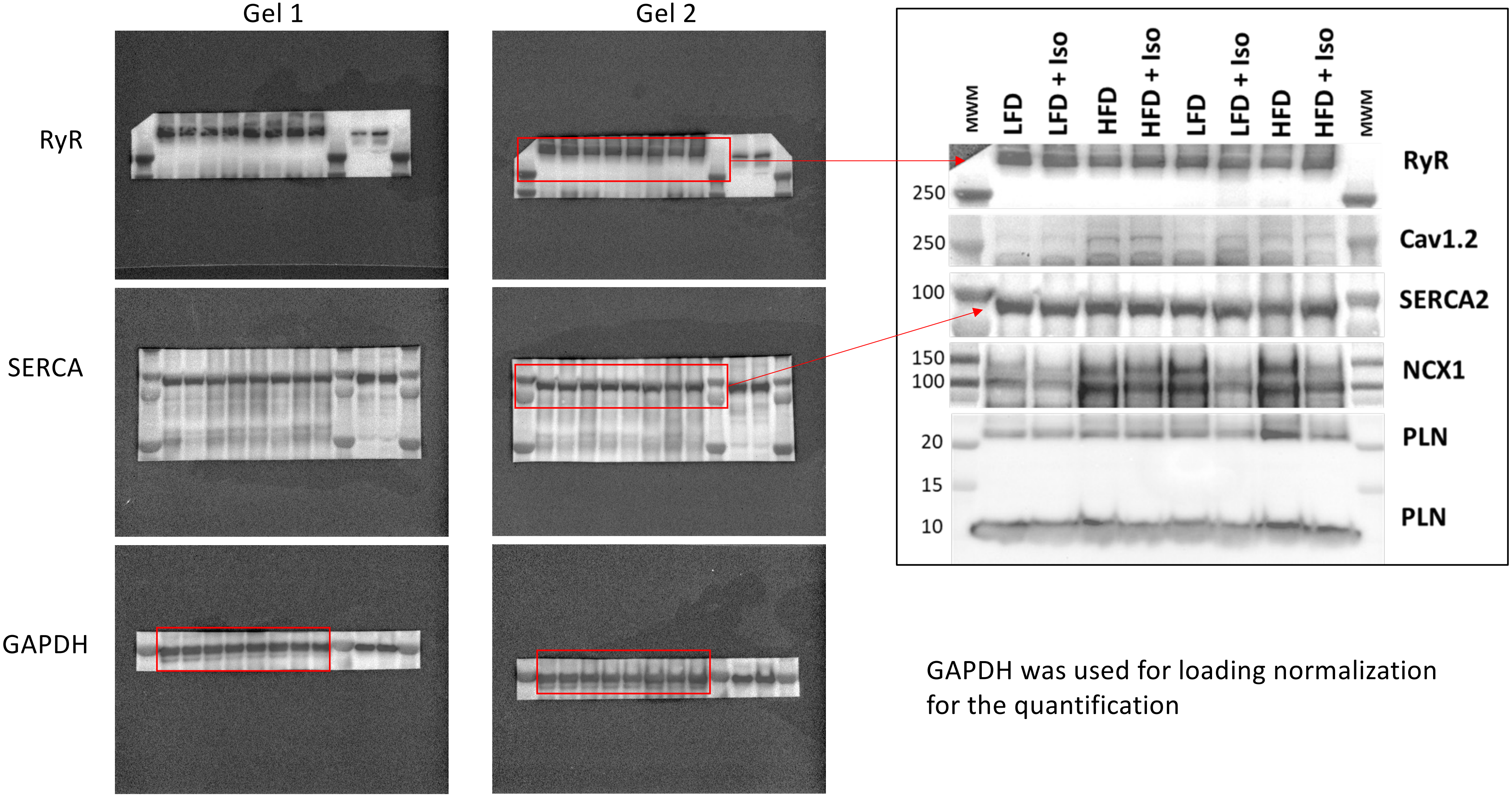

GAPDH was used for loading normalization for the quantification

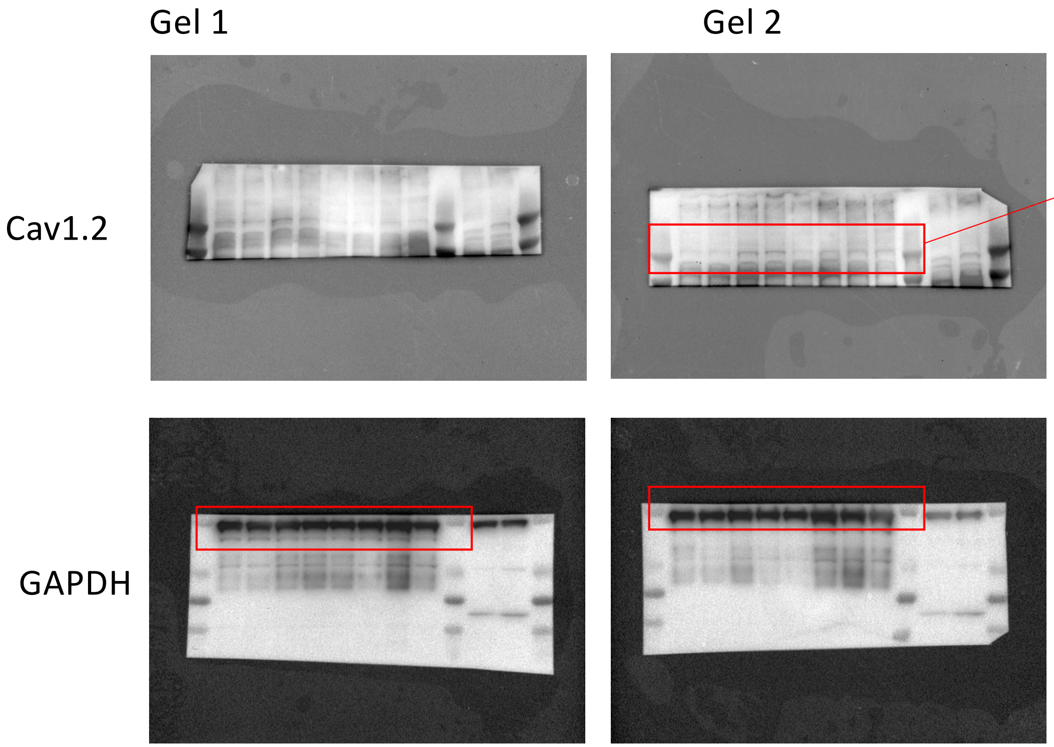

Figure 8A of manuscript

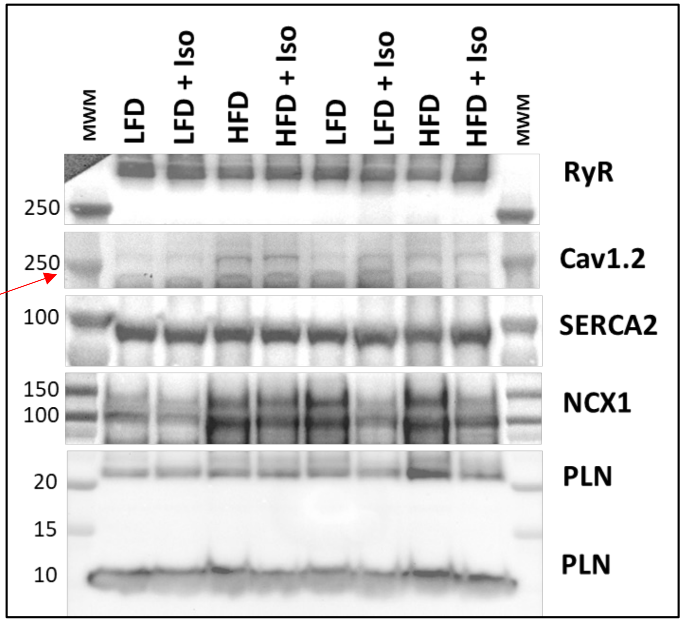

GAPDH was used for loading normalization for the quantification

GAPDH was blotted in the same membrane as PLN

Figure 8A of manuscript

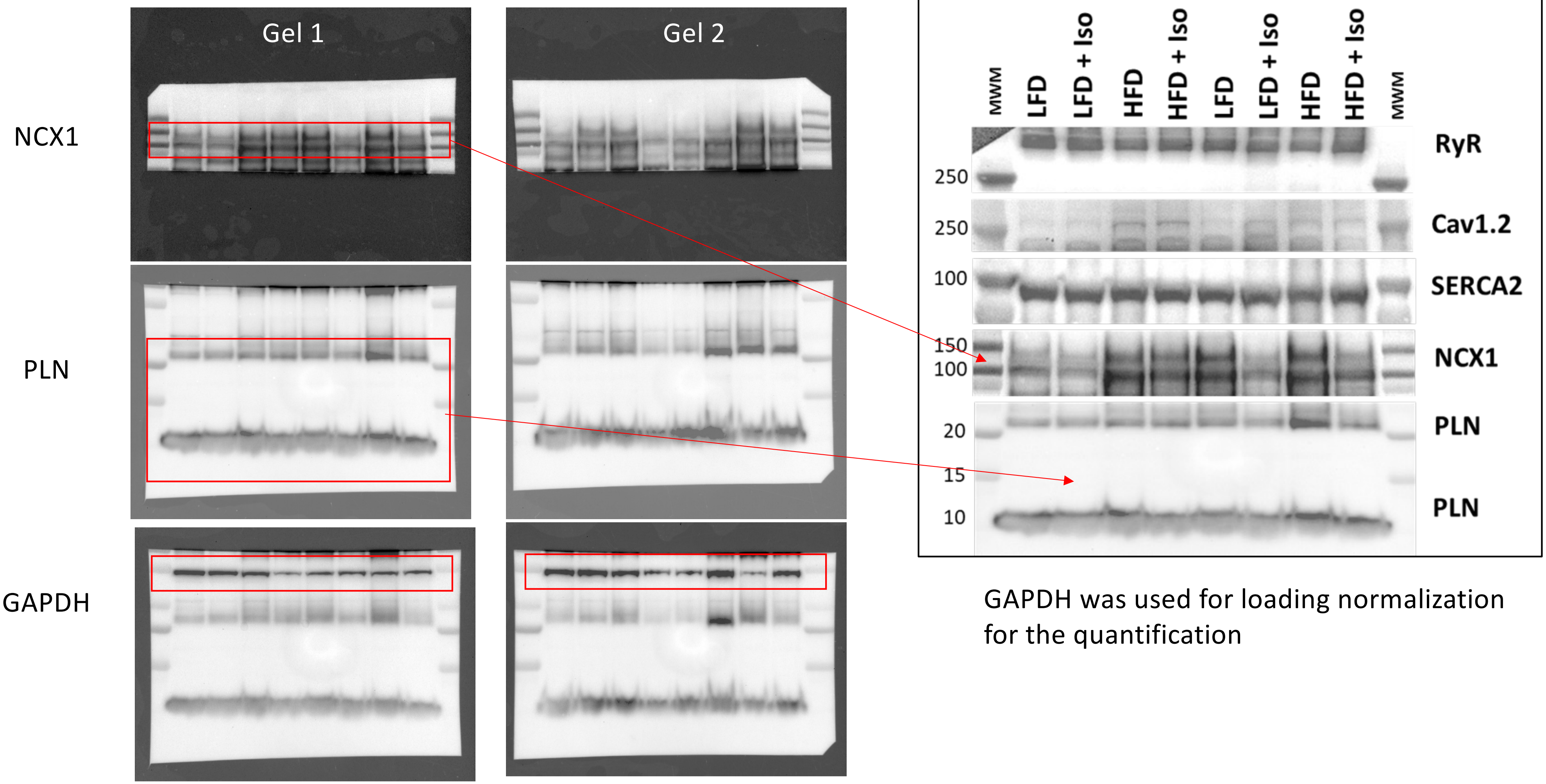

GAPDH was used for loading normalization for the quantification

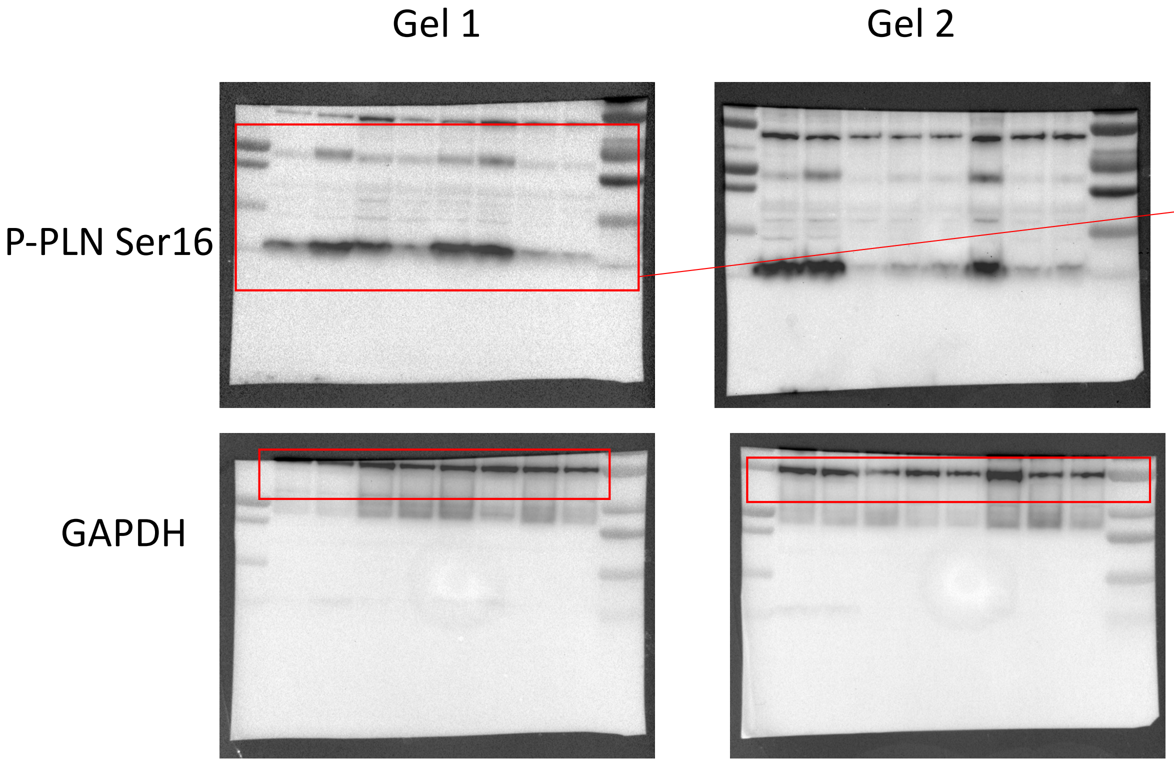

Figure 8C of manuscript

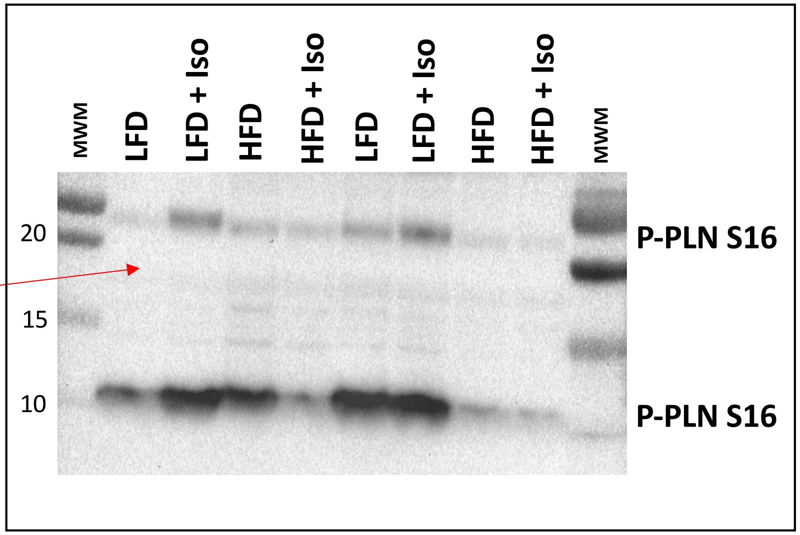

GAPDH was used for loading normalization for the quantification
